# Occurrence of neutrophil dysplasia in the course of severe nephrotic syndrome in a 12-year-old boy on immunosuppressive therapy: Answers

**DOI:** 10.1007/s00467-016-3447-9

**Published:** 2016-06-30

**Authors:** Lidia Hyla-Klekot, Paweł Rajwa, Andrzej Paradysz, Piotr Bryniarski

**Affiliations:** 1 Department of Pediatric Nephrology, Pediatrics and Oncology Center, 41-500 Chorzów, Poland; 2 Department of Urology, Medical University of Silesia, 41-800 Zabrze, Poland

**Keywords:** Pelger–Huët anomaly, Nephrotic syndrome, Immunosuppressive therapy, Mycophenolate mofetil, PPHA

## Answers

1. The changes in granulocyte nuclei morphology along with the presence of immature forms in peripheral blood (after the exclusion of myelodysplastic syndrome, leukemia, and infection) allowed us to diagnose the pseudo Pelger–Huët anomaly (PPHA).
2. A unilobed neutrophil in the patient’s peripheral blood.
3. It was decided to administer another dose of rituximab (Rtx), which resulted in the maintenance of CD20 lymphocyte depletion with subsequent remission of the nephrotic syndrome. This also allowed us to terminate corticosteroid therapy.

## Discussion

Pelger–Huët anomaly (PHA) is a very rare, multi-factorial disorder of granulocytopoiesis that manifests itself in reduced nuclear segmentation and excessive chromatin condensation in neutrophils [1–4]. The disease may be hereditary or acquired. The cause of congenital PHA is probably a mutation in the lamin B receptor (*LBR*) gene [1]. Presumably, in the acquired form of PHA, the pseudo-Pelger–Huët anomaly (PPHA), etiological factors reversibly suppress expression of the *LBR* gene and block the functions of the LBR protein [5]. PPHA may occur after the use of certain medications and in patients who suffer from hematological or infectious disorders [5]. Owing to the fact that dysgranulopoiesis may be a marker of a myelodysplastic or myeloproliferative process, the emergence of granulocyte abnormalities in peripheral blood in a child who has been treated for many years using immunosuppressive drugs, should raise great concern regarding neoplastic processes. In differentiating between proliferative disorder and iatrogenic forms of PPHA, the key elements are: clinical data, reversibility of the process, assessment of peripheral blood smear and bone marrow biopsy [5]. It was impossible for us to establish a final diagnosis without extensive tests. Initially, both the myelogram and the *BCR* mutation genetic test allowed us to reject suspicions of a myeloproliferative process and myelodysplastic syndrome (MDS). Most of the reported cases of PPHA refer to patients after vascularized organ transplantation who receive immunosuppressive polytherapy (mainly mycophenolate mofetil (MMF) as a basic drug in the prevention of transplant rejection) [5, 6]. At the time of the occurrence of dysgranulopoiesis, our patient was at a critical moment of immunosuppressive therapy conversion. In the regimen, the patient was receiving a full dose of MMF. Two weeks prior to the manifestation of PPHA, Rtx was administered due to the high frequency of nephrotic syndrome relapses. Nephrotic syndrome additionally had features of severe steroid and cyclosporine A (CsA) toxicity apparent both clinically and microscopically. The dose of MMF was immediately reduced and subsequently withdrawn after the occurrence of PPHA, which caused the normalization of complete blood count (CBC). It is essential to point out the fact that MMF used in monotherapy did not lead to any morphological granulocyte changes. Perhaps an explanation of the observed phenomenon is the pharmacological potentialization of the effect of given drugs. It may also suggest that MMF, as an inhibitor of inosine-5′-monophosphate dehydrogenase (IMPDH), has a myelosuppressive activity [6]. Presumably, the mechanism leading to the occurrence of PPHA may be associated with drug interactions or impairment of the function of enzymes that are involved in the transport and metabolism of drugs. There are many hypotheses that suggest a possible mechanism of an impaired process of granulocyte nuclei segmentation in PPHA, e.g., individual variation of IMPDH activity, which has an influence on adverse effects of MMF [6]. However, the most probable cause of PPHA seems to be multi-drug interactions [1–5]. Determination of a main role for one of the drugs in particular is almost impossible. The majority of patients described in publications received polytherapy. Despite the fact that in recent brief reports there is some information regarding the possibility of PPHA occurrence after Rtx administration, the most probable cause of the PPHA in this patient was the MMF interactions, since only total withdrawal of MMF resulted in normalization of hematopoietic cells.

## Conclusions

Combination therapy with the use of MMF and other immunosuppressive or antiviral drugs may probably cause dysgranulopoiesis in children who are treated due to transplantation and immune-related diseases. Regarding both the recent report on severe hypogammaglobulinemia incidence and our experiences with granulocyte nuclei deformation after the administration of MMF, it seems rational to understand the effect of MMF as a multi-level immune system suppression, especially in extensively applied polytherapy.

To conclude, the case reminds us that during immunosuppressive therapy it is essential to remain vigilant and cautious, due to the fact that the possible occurrence of blood cell abnormalities may require the differentiation between relatively frequent diseases, such as reactive illnesses, proliferative disorders or myelodysplastic syndromes, and uncommon medical conditions like the PPHA.
